# Hemophagocytic Lymphohistiocytosis and Miliary Tuberculosis in an Apparently Immunocompetent Patient: A Case Report

**DOI:** 10.3390/idr16040058

**Published:** 2024-08-17

**Authors:** Filippo Ducci, Francesca Mariotti, Jessica Mencarini, Claudio Fabbri, Alessandra Francesca Manunta, Daniela Messeri, Paola Parronchi, Pierluigi Blanc, Alessandro Bartoloni

**Affiliations:** 1 Department of Experimental and Clinical Medicine, University of Florence, 50134 Florence, Italy; filippo.ducci@unifi.it (F.D.);; 2 Infectious and Tropical Diseases Unit, Careggi University Hospital, 50134 Florence, Italy; mencarinij@aou-careggi.toscana.it; 3 Unit of Infectious Diseases, San Jacopo Hospital, Azienda ASL Toscana Centro, 51100 Pistoia Pe, Italy; 4 Immunology and Cellular Therapies Unit, Careggi University Hospital, 50134 Florence, Italy; paola.parronchi@unifi.it

**Keywords:** hemophagocytic lymphoistiocytosis, tuberculosis, paradoxical reaction

## Abstract

Hemophagocytic lymphohistiocytosis (HLH) is a serious haematologic condition that can be related to various diseases, including tuberculosis (TB). The patient is a previously healthy 26-year-old man, originally from western Africa, admitted to hospital for fever and weight loss. Given the results of a computed tomography (CT) scan, ocular examination and microbiologic tests, miliary TB with pulmonary, lymph nodal and ocular involvement was diagnosed. Following the introduction of antitubercular treatment (ATT), an increase in inflammation indexes and severe pancytopenia were observed; at this point, the patient presented with six of the eight diagnostic criteria for HLH, and a diagnosis of HLH secondary to TB was raised. Therefore, HLH treatment with a high dose of dexamethasone was started, with a good clinical response. We performed a literature review of TB-related HLH, which shows a high mortality rate. ATT is necessary to ensure patient survival to remove the antigenic driver. Our patient developed HLH after the initiation of ATT as a paradoxical reaction, which may be linked to the release of antigens due to the bactericidal effect of ATT.

## 1. Introduction

Hemophagocytic lymphohistiocytosis (HLH) is a dysfunctional immune disorder characterised by the excessive activation of macrophages and lymphocytes and the overproduction of proinflammatory cytokines, resulting in systemic inflammation and tissue damages [1,2]. Two main forms of HLH are known: primary or familial HLH, usually related to genetic disorders, and acquired HLH, which is secondary to other diseases, such as neoplasms (mainly haematological) and autoimmune/rheumatologic disorders or infectious diseases. The most common infectious causes are viruses like Epstein–Barr virus (EBV) [3]. Tuberculosis (TB) is a communicable disease with a great burden of morbidity and mortality, especially in low and middle-income countries; according to the World Health Organization, 7.5 million people were newly diagnosed with TB in 2022, with 1.3 million deaths worldwide [4]. Italy is a low-TB-burden country, with an estimated incidence of 4.1 per 100,000 people in 2021; about 57% of cases are diagnosed in non-native people, mainly migrants from highly endemic countries [5]. TB exists in a pulmonary and an extrapulmonary form; the most common extrapulmonary sites are lymph nodes, pleura, bone and the central nervous system, although virtually every organ can be involved. Miliary TB is a form of disseminated disease that occurs after the haematogenous spread of bacilli [6]. Active TB is an uncommon cause of HLH, causing 9–25% of cases where HLH is secondary to infection [2]. Here, we report a case of HLH associated with miliary TB (MTB) in an otherwise immunocompetent healthy man.

## 2. Detailed Case Description

The patient was a healthy 26-year-old man born in western Africa who had been living in Italy for five years. He presented to the Emergency Department (ED) of S. Jacopo Hospital in Pistoia, Italy, after about 3 weeks of fever and a history of about five kg weight loss; the results of a chest X-ray (CXR) were normal, and blood tests (BTs) evidenced mild leukopenia and increased C-reactive protein (CRP). He was discharged with antipyretic therapy. Due to the persistence of symptoms, he returned to the same ED after three weeks. CXR was repeated, with evidence of multiple pulmonary micronodules and BT showing persistent leukopenia (white blood cells, WBCs, 3.7 × 10^9^ cells/L) and increased inflammation indexes (CRP 320 mg/L with normal value < 5 mg/L, procalcitonin, PCT, 24 ng/mL with normal value < 0.5 ng/mL, ferritin > 7500 ng/mL with normal value 30–400 ng/mL) and cholestasis indexes (gamma glutamyl transferase, GGT, 420 U/L; total bilirubin, BLR, 2.5 mg/dL). Therefore, he was admitted to the Infectious Diseases Department.

Upon clinical examination, he was alert and oriented, eupneic and pyretic, with a body temperature of 39.5° Celsius (C). He had mild scleral jaundice, palpable neck lymphadenopathies, a tense abdomen and splenomegaly. Upon suspicion of bacterial infection, broad-spectrum antibiotic therapy with piperacillin/tazobactam and vancomycin was initially started (days 1–5 of hospitalisation); several microbiological tests, including a HIV test, were performed and yielded negative results, while interferon gamma release assay (IGRA, QuantiFERON-TB) repeatedly yielded indeterminate results. Chest–abdomen computed tomography (CT) evidenced pulmonary micro-nodularities and multiple lymphadenopathies at thoracic, abdominal, supraclavicular, and latero-cervical levels, associated with splenomegaly (15.5 cm). Upon suspicion of TB, sputum and bronchial aspirate (BA) with microbiological tests for mycobacteria were performed. Additionally, due to blurred vision in the left eye, an ophthalmologic examination with fluorescein angiography raised suspicion of choroidal tuberculoma.

On the seventh day after admission, with a positive result on sputum and BA of polymerase chain reaction (PCR) for *M. tuberculosis* complex with no evidence of rifampicin resistance mutations, we started four-drug antitubercular therapy (ATT) with rifampin (RIF), isoniazid (INH), pyrazinamide (PZA) and ethambutol (EMB). Examination for acid-fast bacilli was negative, and culture exam on the same samples resulted positive for *M. tuberculosis* with complete sensitivity to first-line TB drugs. Moreover, blood cultures were positive for *M. tuberculosis*, while PCR for *M. tuberculosis* complex on stool and urine resulted negative. Together with ATT, steroid therapy (CS, prednisone 25 mg/day) was started. However, anti-TB drugs were discontinued after 5 days due to a further increase in hepatic cytolysis (glutamic–pyruvic transaminase, GPT, up to 700 U/L) and cholestasis indexes (GGT up to 700 U/L, BLR 8 mg/dL), which raised suspicion of drug-induced liver injury (DILI, mixed pattern). Following a 48 h wash-out, ATT was gradually reintroduced, replacing PZA with moxifloxacin (MXF) due to its lower hepatotoxicity and better ocular penetration; subsequently, transaminases and cholestasis indexes gradually decreased.

**Figure 1:**
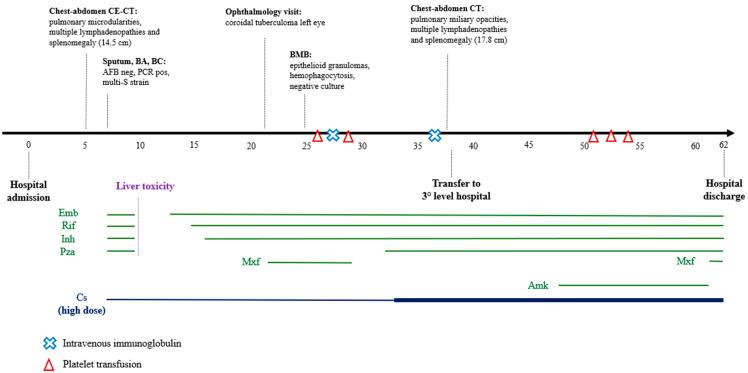
Timeline of radiological and microbiological exams and treatments during hospitalisation. AFB: acid fast bacilli. Amk: amikacin. BA: bronchial aspirate. BC: blood culture. BMB: bone marrow biopsy. CE-CT: contrast-enhanced computed tomography. Cs: corticosteroid. Emb: ethambutol. Inh: isoniazid. Mxf: moxifloxacin. PCR: polymerase chain reaction. Pza: pyrazinamide. Rif: rifampin.

**Figure 2:**
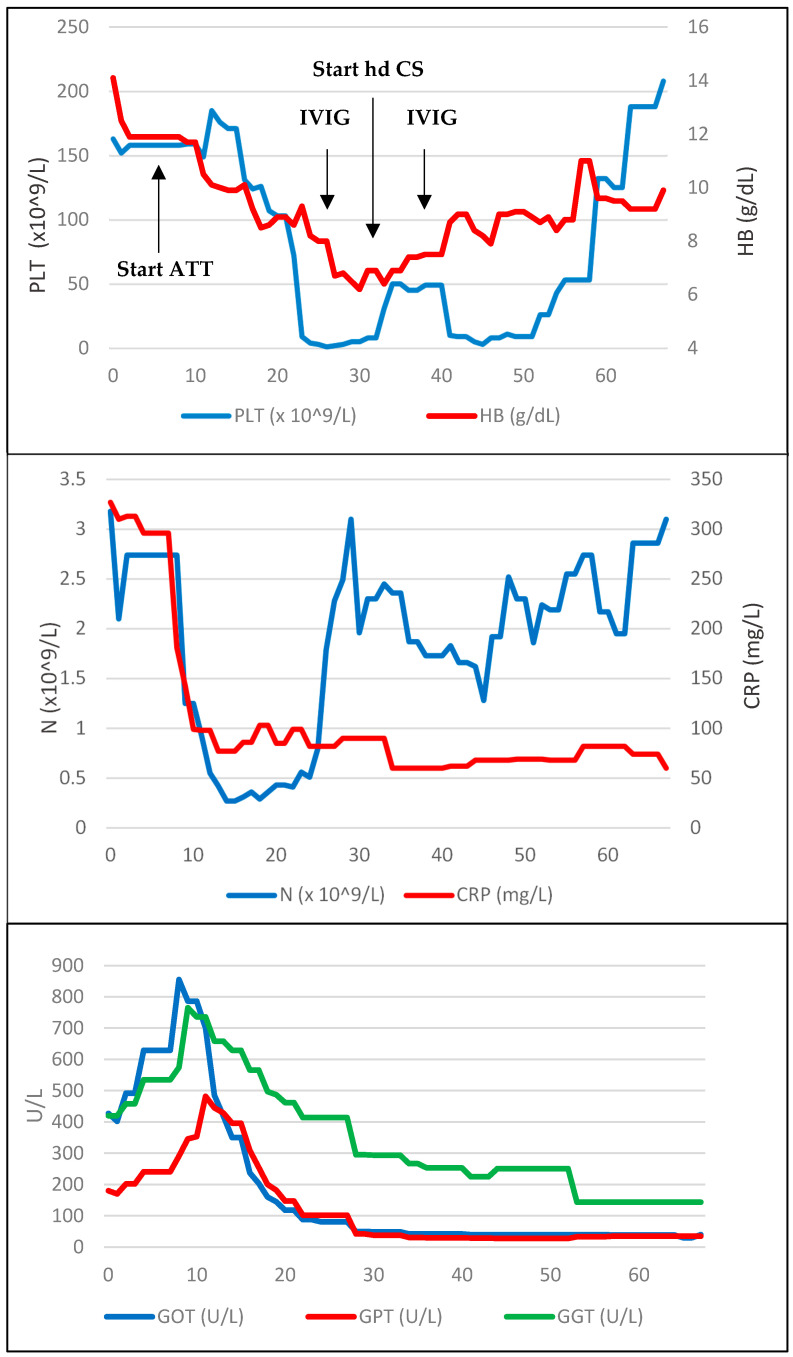
Development of biochemical parameters during the disease course. Day 0 is the first day of hospitalisation. ATT: anti-tuberculous treatment. CRP: C-reactive protein. GGT: gamma glutamyl transferase. GOT: glutamic–oxalacetic transaminase. GPT: glutamic–pyruvic transaminase. HB: haemoglobin. Hd CSs: high-dose corticosteroids. IVIG: intravenous immunoglobulin. N: neutrophils. PLTs: platelets.

Figure 1 shows a summary of the diagnostic and microbiological tests and treatments administered. The trend of biochemical parameters during the course of the disease is summarised in Figure 2.

During this period, clinical conditions did not improve, and the patient was persistently febrile, with daily peaks of more than 39 °C despite ATT. In order to investigate the pathogenesis of pancytopenia, a bone marrow biopsy (BMB) was performed on day 24 of hospitalisation. In the following days, platelet count (PLT) abruptly decreased with a persistently positive Coombs test; this was first attributed to an autoimmune and iatrogenic mechanism related to the introduction of MXF, which was, therefore, discontinued. However, PLT and haemoglobin (HB) counts further decreased (up to 1000 cells/µL and 6.7 g/dL, respectively); thus, PLT transfusion, intravenous immunoglobulin (IVIG, 1 g/kg/day, days 27–28 of hospitalisation) and high-dose CS (dexamethasone 40 mg/day from day 27) were started.

In the following days a clinical improvement was observed, together with the normalisation of body temperature, decreased ferritin values and a slight increase in PLT and HB counts. Histologic examination on BMB showed Langhans-type multinucleated giant cells and epithelioid granulomas, according to the diagnosis of MTB, associated with macrophage activation with aspects of hemophagocytosis. Microbiological culture on BMB was negative for *M. tuberculosis*. At that time, six out of the eight diagnostic criteria for HLH were positive [3] (fever, splenomegaly, trilinear cytopenia, hyperferritinemia, bone marrow hemophagocytosis and decline of natural killer—NK—cells,), the H score was 209 [7] and, thus, a diagnosis of HLH secondary to MTB was made. Therefore, a modified HLH treatment protocol [3] was applied (dexamethasone 10 mg/m^2^/day from day 33 of hospitalisation without etoposide), whereas PZA was reintroduced as the fourth antitubercular drug to ensure the adequate treatment of the underlying disease.

Due to ongoing thrombocytopenia, on day 37, the patient was transferred to a tertiary-level hospital (Careggi University Hospital of Florence, Italy), where TB therapy was implemented with intravenous amikacin (from day 48), while CS therapy was confirmed, with a gradual improvement in clinical and biochemical parameters. In agreement with the immunology and haematology consultants, immunosuppressive therapy was not escalated, given the hypothesis of immune reconstitution (IRIS-like picture) elicited by TB therapy and/or potent immune activation related to the high burden of released antigens due to the presence of multi-organ infectious disease. The patient was discharged after a total of 62 days of hospitalisation in good clinical condition with ATT (RIF-INH-EMB-MXF) and CS (prednisone 50 mg/day in tapering regimen) treatment.

After discharge, the patient continued ATT and CS treatment with regular follow-up visits and good clinical course. Blood counts and hepatic function indexes were persistently within a normal range, even after the discontinuation of CS therapy at ninety days after discharge. Immunophenotype on peripheral blood was normal, with a mild decrease in CD3-CD16+CD56+ NK-cells (70/μL, normal 100–200), able to produce interferon (IFN)-γ after polyclonal stimulation (48.5% positive cells; normal range 50–96.7%) and conserved percentages of the other subpopulation of immune cells and normal percentages of interleukin (IL)-17, Tumor Necrosis Factor (TNF)-α and IL-2-producing T cells. A total body CT performed three months after the beginning of treatment showed a reduction in pulmonary micronodularities, abdominal lymphadenopathies and splenomegaly. The patient’s condition continuously improved, and ATT was discontinued after a total of twelve months.

## 3. Discussion

Secondary HLH is a serious haematologic condition elicited by immune hyperactivation in response to different triggers, including autoimmune and autoinflammatory diseases, persistent infections, and malignancies [1]. Different infectious agents have been associated with HLH, such as EBV, Cytomegalovirus, HIV and, most recently, SARS-CoV-2 among viruses and Leishmania among protozoans; among bacteria, the most common causes include Mycobacteria, Rickettsiae and other tick-borne pathogens like Ehrlichia and Anaplasma. These infections can trigger hyperactivation of macrophages and CD8+ T lymphocytes, with an overproduction of proinflammatory cytokines [8,9,10,11].

To the best of our knowledge, two systematic reviews were recently published about the association between TB and HLH; among the 116 cases described, more than half of the patients did not have any comorbidity, and the overall mortality rate was 45%, higher in patients aged under 30 years. Interestingly, the authors evidenced that all patients who did not receive ATT had an unfavourable outcome. The overall mortality rate was higher compared to patients with other infection-induced HLH (generally below 25%) [2,11,12,13]. However, it should be considered that many of the TB-associated HLH cases described in the literature were published before the publication of the HLH management guidelines of 1994 [14] and 2004 [3]; therefore, these patients may have received less effective treatments.

According to Kurver et al., a high proportion of patients with TB-triggered HLH have extrapulmonary localisations of TB, particularly bone marrow involvement [12]. In our patient, *M. tuberculosis* was isolated from respiratory specimens and blood cultures; there was also clinical evidence of eye involvement, although microbiological confirmation from this site was not feasible. Bone marrow biopsy showed a histological pattern consistent with TB, without any isolation of *M. tuberculosis*.

Most patients with TB-associated HLH are anergic, which means the absence of IFN-γ–mediated T-cell memory [12]. As IFN-γ is mainstream in the defence against intracellular bacteria, failure in its production results in defective infection control, as evidenced by the high number of disseminated forms. Thus, persistent antigenic stimulation at a high level may overstimulate the immune system, thus contributing to HLH development.

Diagnosis of HLH is based on criteria applied in the HLH-2004 [3]. Our patient fulfilled six out of the eight criteria (namely, fever, splenomegaly, trilinear cytopenia, hemophagocytosis in bone marrow, reduction in NK-cells and hyperferritineamia); the level of soluble CD25 was not assessed because it was not performed by our laboratory. In addition, the H score was 209, conferring a probability of 88–93% in terms of HLH diagnosis [7].

Tuberculosis-associated HLH diagnosis can be challenging because HLH and MTB have several non-specific, overlapping characteristics, particularly fever, splenomegaly and anaemia; thrombocytopenia and markedly increase in ferritin levels are less common in TB but can still be observed [8]. In our case, PLT counts rapidly dropped down, which is unusual in TB and prompted us to a further evaluation, which led to a HLH diagnosis.

Treatment protocols for HLH are described in 1994 [14] and 2004 guidelines [3] and are based on high-dose intravenous CS, IVIG and immunosuppressive drugs like cyclosporin and etoposide in refractory cases. However, these protocols are mainly validated for familial HLH, while in secondary HLH, a balance between disease-specific therapy and HLH therapeutic protocols is often required [13]. Nevertheless, in TB-HLH, a prompt start of ATT is of paramount importance: in studies conducted by Fauchald and Kurver, the mortality rate was 100% in patients who did not receive ATT, suggesting that the removal of the mycobacterial antigenic drivers is crucial to interrupting immune hyperactivation [2,12].

Our patient developed HLH when a TB diagnosis had already been established, and he was already on ATT, while at the time of hospital admission, the HLH criteria were not fulfilled. There are reports of patients who developed TB-HLH during ATT as a paradoxical reaction (PR) [12]. Paradoxical reaction is defined as the worsening of pre-existing tuberculous lesions or the appearance of new lesions despite the initial improvement of clinical symptoms with ATT. Although the pathogenesis of PR is not clarified, it might be linked to an excessive release of mycobacterial antigens by dying infected macrophages [15]. In addition, as in our case, the release of antigens due to the bactericidal effect of ATT may have further stimulated the immune system, thus contributing to the development of HLH.

## 4. Conclusions

In conclusion, this case emphasises that HLH should be considered in patients with MTB, especially in case of paradoxical worsening during ATT, and timely diagnosis and treatment are crucial to increase the chances of survival. Cytopenia and markedly increased levels of serum ferritin may help to distinguish TB-associated HLH from other conditions.

## Data Availability

No new data were created or analyzed in this study. Data sharing is not applicable to this article.
